# Integrative Multiomics Analysis Identifies HK2 as a Key Regulator of Metabolic Reprogramming in Hepatic Stellate Cells

**DOI:** 10.1155/humu/1584910

**Published:** 2025-11-07

**Authors:** Lu Han, Fan Lu, Shaojie Chen, Qingxiu Zhang, Huayue Wu, Tao Huang, Hongfei Pu, Jinglin Wang, Gaoliang Zou, Chen Pan, Xueke Zhao

**Affiliations:** ^1^Department of Infectious Diseases, The Affiliated Hospital of Guizhou Medical University, Guiyang, Guizhou, China; ^2^Department of Comprehensive Ward, The Affiliated Hospital of Guizhou Medical University, Guiyang, Guizhou, China; ^3^Department of Obstetrics, The Affiliated Hospital of Guizhou Medical University, Guiyang, Guizhou, China; ^4^Department of Hepatobiliary Surgery, The Affiliated Hospital of Guizhou Medical University, Guiyang, Guizhou, China; ^5^Department of Critical Care Medicine, Guizhou Provincial People's Hospital, Guiyang, Guizhou, China; ^6^Department of Gastroenterology, Guizhou Provincial People's Hospital, Guiyang, Guizhou, China; ^7^Department of Gastroenterology, The Affiliated Hospital of Guizhou Medical University, Guiyang, Guizhou, China

**Keywords:** aerobic glycolysis, hepatic stellate cells, HK2, liver fibrosis, RNA sequencing, single-cell sequencing, therapeutic target, WGCNA

## Abstract

**Background:**

Liver damage caused by chronic liver disease frequently leads to hepatic fibrosis. A pivotal step in the fibrotic process is the activation of hepatic stellate cells (HSCs). Previous studies have suggested that enhanced aerobic glycolysis is closely associated with HSC activation. However, a comprehensive analysis of the relationship between hepatic fibrosis and aerobic glycolysis remains lacking.

**Methods:**

RNA sequencing of liver tissue from 30 patients with fibrosis or cirrhosis and 8 healthy controls was conducted as part of a comprehensive multiomics approach to discover differentially expressed genes (DEGs). Weighted gene coexpression network analysis (WGCNA) was conducted to detect gene modules associated with liver fibrosis. Functional analyses, including migration and wound healing, were subsequently performed. Furthermore, a machine learning model predicting fibrosis was constructed based on glycolysis-related gene expression and validated using an independent dataset. Its clinical significance was subsequently explored. Protein expression and localization were further validated via western blotting and immunohistochemistry techniques.

**Results:**

The expression of HK2 is notably increased in HSCs and is strongly linked to the advancement of liver fibrosis. Within the constructed machine learning model, the random forest algorithm demonstrated the highest predictive performance for liver fibrosis, achieving an area under the curve (AUC) of 0.889. HK2 expression levels also had a positive correlation with clinical signs of liver damage, such as ALT and AST levels. Knockdown of HK2 in HSCs markedly impaired their migratory capacity and wound healing ability.

**Conclusions:**

HK2 is involved in activating HSCs, thus promoting the progression of liver fibrosis. These findings suggest that HK2 holds potential as a therapeutic target for liver fibrosis and as a biomarker for predicting its progression.

## 1. Introduction

Liver fibrosis is a chronic liver condition caused by infectious pathogens such as hepatitis B and C viruses, as well as noninfectious diseases including nonalcoholic fatty liver disease [1, 2]. When the liver is continuously subjected to inflammatory irritation, the extracellular matrix accumulates excessively; this stage is known as liver fibrosis [3, 4]. In some cases, this condition may advance to cirrhosis or even hepatocellular carcinoma [5]. Aerobic glycolysis plays a major role in liver fibrosis, which is triggered by hepatic stellate cell (HSC) activation [6–10]. Despite this, effective targeted therapies for liver fibrosis remain unavailable [11].

Research has demonstrated that aerobic glycolysis is a critical metabolic feature during the transdifferentiation of quiescent HSCs [7, 10]. The activation of HSCs requires a constant energy supply to support their proliferative activity [9, 12]. Studies previously conducted have revealed that inhibiting aerobic glycolysis can delay the progression of liver fibrosis [13]. Hexokinase 2 (HK2) is a key enzyme in glycolysis, facilitating the conversion of glucose into glucose-6-phosphate (G6P). By interacting with VDAC1 on the outer mitochondrial membrane, HK2 promotes ATP synthesis and prevents apoptosis [14]. Prior findings suggest that HK2 aggravates liver fibrosis in mice [15]. The targeted deletion of HK2 was shown to effectively curb liver fibrosis in both in vivo and in vitro conditions, suggesting HK2 as a viable therapeutic target for liver fibrosis [16]. However, a comprehensive analysis of the role of HK2 in human liver fibrosis tissue remains incomplete.

In this study, we integrated multiomics analyses with experimental validation, applying differential expression analysis, WGCNA, and clinical correlation approaches to ultimately identify HK2 as a key gene of interest. Our results suggest that HK2 is central to the pathogenesis of liver fibrosis and may be valuable as a therapeutic target and biomarker. Single-cell transcriptomic analysis further revealed that HK2 is predominantly expressed in HSCs, a result corroborated by western blotting (WB) of human and murine liver tissues. Additionally, HK2 knockout (KO) and knockdown experiments provided mechanistic insights, demonstrating its critical influence on HSC migration and wound healing processes.

## 2. Methods

### 2.1. Reagents and Antibodies

CCl_4_ and corn oil were acquired from Sigma-Aldrich (St. Louis, Missouri, United States). The antibodies used in this study were mouse anti-*β*-actin (dilution 1:1000) and rabbit anti-HK2 (dilution 1:1000), which were sourced from Chengdu Zhengneng. The Masson staining kit, HE staining kit, and Sirius Red staining kit were purchased from Beijing Solembo, while total protein extraction kits (KTP3006) were provided by Abbot Gold (China).

### 2.2. Participant Recruitment and Clinical Data Collection

Human liver tissue was acquired from donors under the guidelines of the Helsinki Declaration, with the Clinical Trial Ethics Committee of the Affiliated Hospital of Guizhou Medical University approving (Approval Number: 2023 lunshen 401). Healthy livers originate from other surgeries, such as distal livers from hepatic hemangiomas or rupture injuries. After HE staining, the pathologists decided to classify them as normal liver tissue. Patients aged 18–70 who had been diagnosed with cirrhosis from various etiologies were included in the study conducted at our hospital. Initial diagnoses were made via imaging and subsequently confirmed by two pathologists through histopathological examination. All participants gave informed consent and underwent a liver biopsy. Exclusion criteria included diabetes, hepatic glycogen storage diseases, and other conditions impacting glucose metabolism. Patients who had not taken insulin or hypoglycemic drugs in the preceding 3 months and those without thyroid or other metabolic disorders were included. With the Ethics Committee of the Affiliated Hospital of Guizhou Medical University giving their approval and all participants providing signed informed consent, a total of 38 individuals were recruited for the study. The sample for transcriptomic analysis consisted of 30 patients with liver fibrosis/cirrhosis and 8 healthy controls. Questionnaires and medical records were used to document traditional risk factors for liver fibrosis/cirrhosis, as well as potential confounders such as age, gender, total bilirubin, total bile acids, alanine aminotransferase, aspartate aminotransferase, and hepatitis B virus infection. Cirrhosis was diagnosed through laboratory tests and histopathological examinations, which were essential for clinical evaluation.

### 2.3. RNA Extraction

To remove genomic DNA contamination, an RNA purification kit was employed. A Bioanalyzer Agilent 2100 or an equivalent device was used to verify the integrity of the samples (RIN ≥ 7.0). The total RNA was then processed using either poly(A) tail enrichment or rRNA depletion techniques to isolate mRNA or noncoding RNA fragments. Following reverse transcription of the RNA into cDNA, the next steps included PCR amplification and library construction, with sequencing adapters being added to the cDNA fragments. Quality control was conducted on the libraries, and after measuring the library concentration and analyzing fragment lengths, the samples were prepared for high-throughput sequencing.

### 2.4. Data Acquisition

Four publicly available datasets were obtained from the GEO database: GSE197112, GSE252249, GSE139602, and GSE175939. GSE197112 contains gene expression data from four normal liver tissue samples and four hepatic fibrosis samples. GSE139602 includes transcriptome data from 5 fibrosis cases, 8 compensated cirrhosis cases, 12 decompensated cirrhosis cases, and 6 healthy controls. GSE229154 contains transcriptome data on three healthy mouse primary liver fibroblasts, three activated mouse primary liver stellate cells, and three activated mouse primary liver stellate cells from HK2-deficient mice. The dataset GSE175939 includes single-cell RNA sequencing data from primary HSCs of a healthy mouse and a mouse treated with CCl_4_. The normalized expression matrix was used for subsequent analyses after the data were downloaded and parsed using the GEOquery package [17]. Single-cell sequencing data was preprocessed using the Seurat package, including normalization, downscaling, and clustering analysis.

### 2.5. Differential Expression Analysis

All analyses were performed using R software (Version 4.3.1). The edgeR package was employed to normalize read counts and identify differentially expressed genes (DEGs). DEGs were defined as those exhibiting an adjusted *p* value < 0.05 and an absolute log2 fold change > 1.5. To control the Type I error rate arising from multiple comparisons, the Bonferroni correction was applied to adjust the threshold of statistical significance.

### 2.6. WGCNA for Identifying Clinically Relevant Gene Modules

Gene modules associated with clinical traits were identified through the application of WGCNA [18]. The process first filters out genes with low expression, then constructs a correlation matrix based on gene expression data, and subsequently converts it into an adjacency matrix to reflect the strength of gene coexpression associations. After the adjacency matrix was made, a topological overlap matrix (TOM) was developed, and hierarchical clustering was employed to create a gene dendrogram. Dynamic tree-cut algorithms were used to identify gene modules. An eigengene and its first principal component were computed for each module to evaluate their relationship to clinical traits. Modules showing strong correlations with the traits of interest underwent further functional enrichment analysis.

### 2.7. Analyses of Gene Ontology (GO) and Kyoto Encyclopedia of Genes and Genomes (KEGG) Enrichment

To determine the biological processes, cellular components, molecular functions, and pathways associated with the identified gene sets, GO and KEGG enrichment analyses were conducted [17–19]. The clusterProfiler R package was used to perform these enrichment analyses. The identification of enriched GO terms and KEGG pathways was based on an adjusted *p* value (Benjamini–Hochberg method) of less than 0.05 [19]. The results were visualized through dot plots, highlighting the top enriched terms and pathways.

### 2.8. Mfuzz Analysis

The Mfuzz package was utilized to cluster and analyze gene expression data in this study. A filtering process was applied after normalizing the gene expression matrix. To ensure effective clustering, the optimal fuzzification parameter (*m*-value) was determined using the mestimate function. To classify genes with similar expression patterns into distinct clusters, the fuzzy C-means algorithm was used, and the number of clusters (*c*-value) was determined. Ultimately, the expression trends of individual gene clusters were assessed, and functional enrichment analysis was carried out to discover any potential biological implications.

### 2.9. Ninety-Four Genes Analyzed for Correlation With Aerobic Glycolysis Genes

The correlation between 94 target genes and aerobic glycolysis genes was analyzed by obtaining the gene set for the aerobic glycolysis pathway from the GSEA website. This gene set comprises core genes involved in aerobic glycolysis. Expression data for 94 target genes were analyzed against the aerobic glycolysis gene set. The analysis employed Spearman's correlation coefficient to assess nonlinear correlations [20]. The analysis was conducted in R using the cor() function, specifying the method as “Spearman” to calculate the gene correlations. Significant correlation results were visualized in heatmaps [21].

### 2.10. Examination of the Top 10 Genes Shows the Strongest Correlation With Glycolysis and Clinical Traits

Prior to investigating the correlation between the Top 10 genes and clinical biochemical indices, we identified the genes most significantly correlated with aerobic glycolysis. A Spearman correlation analysis was performed with the gene expression data and clinical biochemical indices (AST, ALT, and ALP) to investigate any nonlinear relationships. The cor() function in R was utilized for the analysis, with the method specified as “Spearman.” Finally, the results were visualized as heatmaps, illustrating the strength and direction of the correlations between genes and biochemical indices.

### 2.11. Machine Learning

Lasso regression, random forest, logistic regression, support vector machine, and XGBoost were employed to predict liver fibrosis, using the Top 10 genes most linked to aerobic glycolysis [19, 22]. Liver fibrosis occurrence served as the outcome variable. The model was trained and evaluated through cross-validation to ensure stability and generalizability. The diagnostic performance of each gene was then assessed individually using ROC curves and AUC values to evaluate their ability to predict liver fibrosis. Analyses were conducted using R (Version 4.3.1), employing the ggplot package to visualize model performance and the diagnostic efficacy of each gene. A total of 38 transcriptome sequencing data were used as internal data for model training, with 80% of the data being divided into the training set and 20% into the test set. Finally, the data downloaded from GEO was merged for external validation. Eighty percent of the data was divided into the training set and 20% into the test set. The model was validated using external datasets.

### 2.12. Single-Cell Analysis

The single-cell RNA sequencing data of mouse HSCs from the GEO database (GSE175939), including samples from both healthy and CCl4-treated conditions, were analyzed to investigate the role of HK2 in liver fibrosis. After removing housekeeping genes, quality control and normalization were performed to ensure data integrity [19]. Dimensionality reduction was conducted using principal component analysis (PCA) based on highly variable genes. Unsupervised clustering was applied to identify distinct cell populations, followed by detailed cell-type annotation using the SingleR package and manual curation. Differential expression analysis was conducted to compare gene expression between healthy and CCl4-treated HSCs using DESeq2. HK2-expressing cells were identified, and their proportions across different cell types were analyzed to determine their potential involvement in liver fibrosis. The findings provide insights into the cellular distribution and regulatory role of HK2 in hepatic fibrosis, highlighting its potential as a therapeutic target.

### 2.13. Animal Model Preparation

Ten male C57BL/6J mice were randomly assigned to the control or CCl4 treatment groups. The mice were housed under controlled conditions at Guizhou Medical University's animal breeding facility, with a stable temperature of 23°C–25°C, a 12-h light/dark cycle, and humidity maintained at 55% ± 5%. All mice had free access to water and standard chow, and there was a 7-day acclimatization period before the start of the experiment. Control mice were intraperitoneally injected with peanut oil, and mice in the CCl4 group were intraperitoneally injected with 10% CCl4 (15 *μ*L/g) three times a week for 6 weeks. Body weights were consistently monitored and recorded weekly throughout the experiment. Plasma samples and liver tissues were collected. The study was approved by the Animal Ethics Committee of Guizhou Medical University (No. 2403086), and all methods were performed in accordance with relevant guidelines and regulations.

The mice were fasted for a day and then anesthetized by administering pentobarbital intraperitoneally at a dosage of 50 mg/kg. After the mice were deeply anesthetized, they were humanely euthanized by cervical dislocation according to the American Veterinary Medical Association (AVMA) euthanasia guidelines, following the experimental protocol. Posteuthanasia tissues were then collected.

### 2.14. WB

The Abbkine protein extraction kit was used to extract proteins, and their concentrations were determined by the BCA assay. The same quantity of protein was separated via SDS-PAGE and then transferred onto a PVDF membrane. After blocking the membrane with 5% nonfat milk in TBST, it was left to incubate overnight at 4°C with primary antibodies and subsequently with HRP-conjugated secondary antibodies. Protein bands were visualized with an ECL detection system and quantified using ImageJ software for densitometry, with *β*-actin serving as the loading control.

### 2.15. Immunohistochemistry

Liver tissue sections embedded in paraffin were processed for immunohistochemistry. Antigen retrieval in citrate buffer (pH 6.0) was conducted using a microwave after deparaffinization and rehydration were completed. The application of 3% hydrogen peroxide was used to block endogenous peroxidase activity. The tissue sections were incubated with primary antibodies overnight at 4°C and then with HRP-conjugated secondary antibodies. The signal was detected using a DAB substrate, and the sections were counterstained with hematoxylin. The images were obtained with a light microscope, and the staining intensity was calculated using ImageJ software.

### 2.16. Cell Culture

LX-2 cells obtained from Wuhan Procell were grown in high-glucose DMEM supplemented with 10% FBS and 1% penicillin–streptomycin and maintained at 37°C in a 5% CO_2_ incubator. When the cells reached 80% confluence, they were washed with PBS, treated with 0.25% trypsin for digestion, and then neutralized using medium containing FBS.

### 2.17. Transfection and Small Interfering RNA (siRNA) KO

GenePharma in Shanghai, China, provided the siRNAs and nonspecific control siRNAs. Once LX-2 cells reached 50%–60% confluence in 6-well plates, the DMEM medium was switched out for Opti-MEM. A mixture of 10 *μ*L siRNAs (20 *μ*M) and 5 *μ*L liposome transfection reagent was added to 2 mL of Opti-MEM and gently mixed with the cells. After a 6-h period, the medium was exchanged for fresh growth medium. The siRNA sequences were as follows:

HK2: 5′-GGG GAUGAAG GUAGAAUTT-3′

Negative control (NC): 5′-UUCUCCGAACGUGUCACGUTT-3′

### 2.18. Wound Healing

The process of wound healing assays was carried out in 6-well plates with LX-2 cells. The cells were scratched with a 10-*μ*L pipette tip after reaching 90% confluence. The debris was removed, and the wells were washed with PBS. A medium with 1% FBS was added to support migration. Images were taken at 0 and 72 h to assess wound closure and cell migration capacity.

### 2.19. Transwell

The Transwell migration assays were performed using 24-well plates and Transwell chambers equipped with 8 *μ*m pores. LX-2 cells were seeded into each chamber at a concentration of 2 × 10^4^ in serum-free DMEM, with the lower wells containing 600 *μ*L of DMEM with 20% FBS to act as a chemoattractant. The plates were incubated at 37°C with 5% CO_2_ for a period of 6 h. Nonmigrated cells were removed with a cotton swab after incubation. After the cells had migrated to the lower surface, they were fixed with 4% paraformaldehyde for 10 min, stained with 0.1% crystal violet for 20 min, and then rinsed with PBS. The migrated cells were then observed and counted using a microscope.

### 2.20. Proteomic Analysis

Liver tissue proteins were extracted, quantified using the BCA assay, and digested with trypsin. Peptides were analyzed by LC-MS/MS on a Thermo Fisher Q Exactive HF mass spectrometer. The data were processed with MaxQuant, and differentially expressed proteins were identified based on LFQ intensity (fold change > 1.5, *p* < 0.05).

### 2.21. Statistical Analysis

GraphPad Prism software (Version 9.0) and ImageJ were used for statistical analysis. Student's *t*-tests were utilized to compare two groups with normally distributed data. For multiple comparisons, the Bonferroni correction was applied to adjust the significance level. The mean ± standard deviation is used to present the results, with statistical significance set at *p* < 0.05 for all comparisons.

## 3. Results

### 3.1. Gene Expression Profiling and Identification of DEGs in Liver Fibrosis

**Figure 1:**
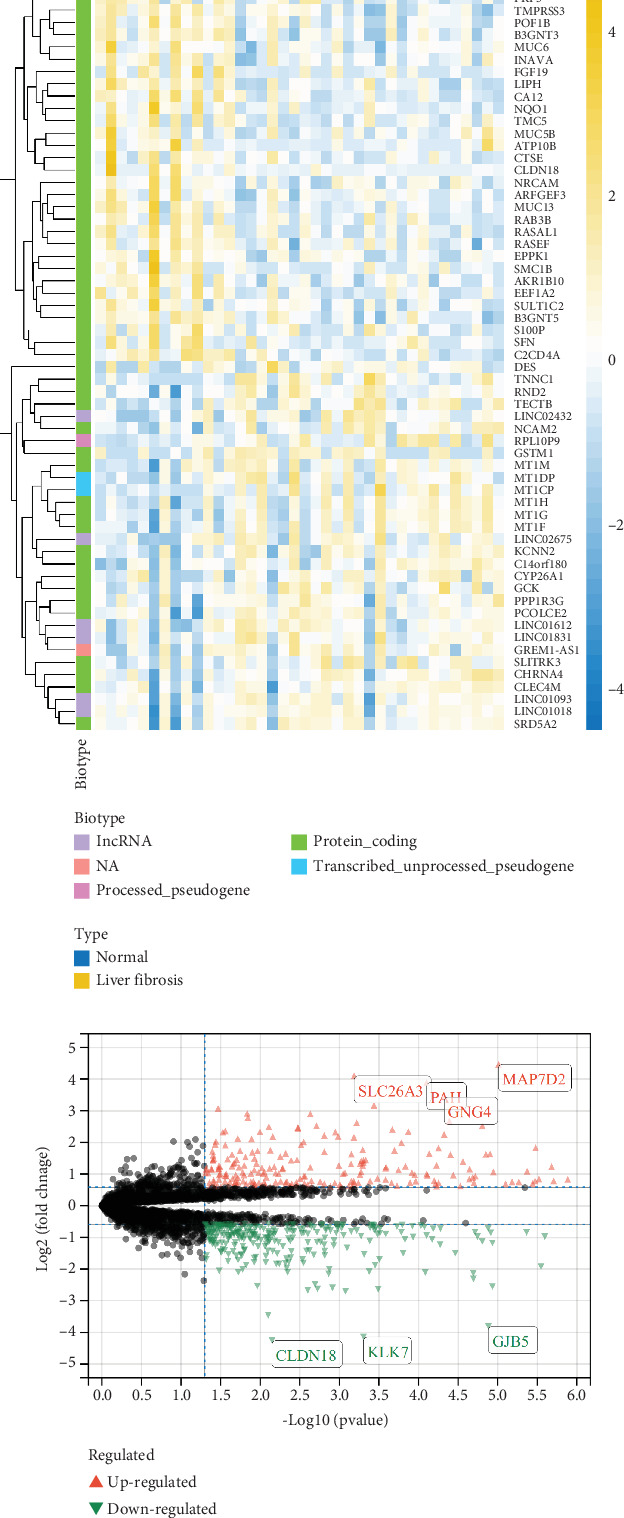
Differential gene expression analysis. (a) Heatmap of differentially expressed genes (DEGs) between patients with liver fibrosis and controls, showing clear separation between the groups. (b) Volcano plot highlighting significantly upregulated (red) and downregulated (green) genes, including notable examples such as SLC26A3 and CLDN18.

Liver tissue samples were collected from 30 patients with liver fibrosis/cirrhosis and 8 healthy livers (Table S1). RNA sequencing analysis was performed on liver fibrosis and control groups, and heatmaps of DEGs (Figure 1a) revealed expression patterns between the two groups, while volcanic maps (Figure 1b) showed significantly upregulated (red) and downregulated (green) genes. Notable upregulated genes include SLC26A3, PAH, GNG4, and MAP7D2, while downregulated genes include CLDN18, KLK7, and GJB5.

### 3.2. Identification of Key Gene Modules by WGCNA

**Figure 2:**
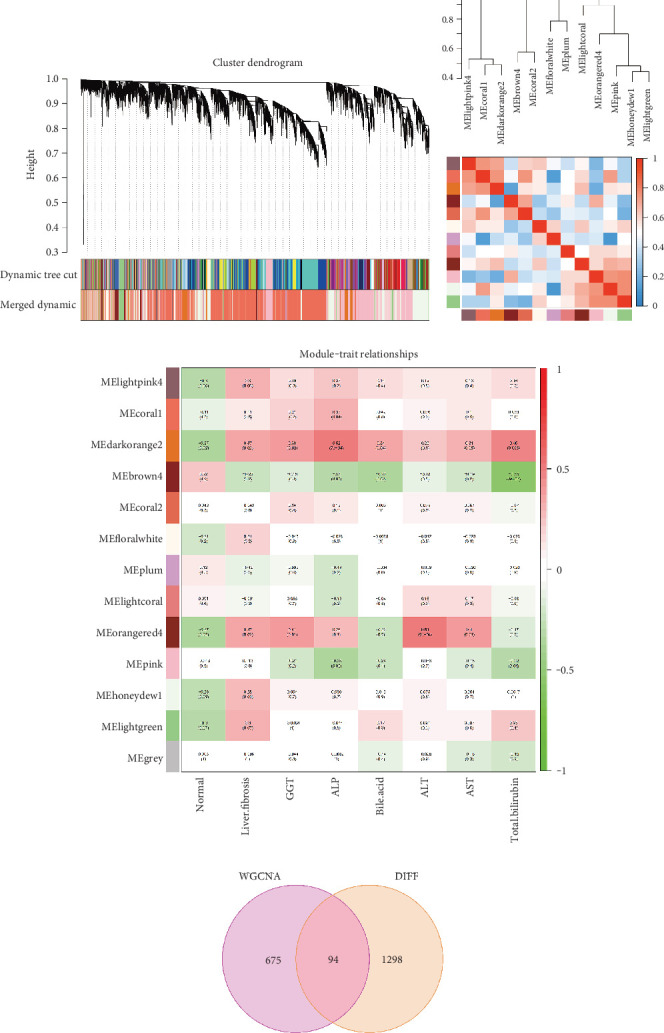
WGCNA and module–trait relationships. (a) Hierarchical clustering dendrogram from WGCNA used to identify coexpression modules. Modules were detected via dynamic tree cutting and merged based on similarity. (b) Eigengene dendrogram and heatmap showing relationships among modules. (c) Heatmap of module–trait correlations, with positive correlations in red and negative in green. (d) Venn diagram displaying 94 overlapping genes between WGCNA-identified modules and DEGs.

To further identify the association between genes and clinical modules, we proceeded with WGCNA analysis (Figure 2a). The heatmap of module correlations (Figure 2b) reveals that the lightpink module exhibits the strongest correlation with the darkorange2 module. The darkorange2 module had a positive correlation with liver fibrosis (*r* = 0.37, *p* = 0.07, Figure 2c), a negative correlation with the control group, and a positive correlation with other clinical traits. Hence, darkorange2 was selected for further analysis. After intersecting the 675 genes in the darkorange2 module with DEGs, a total of 94 genes were identified for further analysis (Figure 2d).

### 3.3. Functional Enrichment Examination

**Figure 3:**
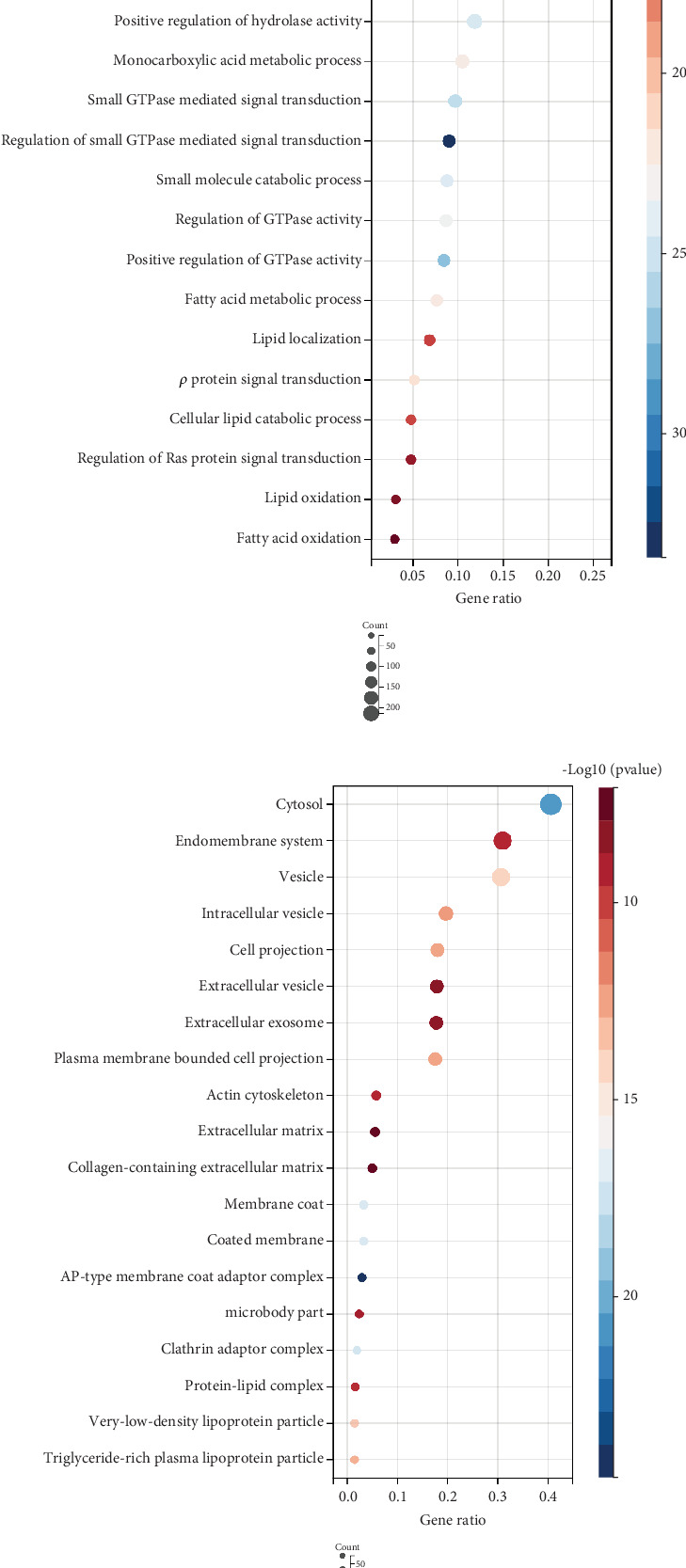
Functional enrichment analysis of genes from the darkorange module. (a) KEGG pathway enrichment analysis highlighting significant pathways. (b) Gene Ontology (GO) biological process (BP) enrichment analysis. (c) GO cellular component (CC) enrichment analysis. (d) GO molecular function (MF) enrichment analysis.

Functional enrichment analysis of DEGs from the darkorange2 module indicated significant enrichment in a number of pathways and biological processes. The KEGG pathways (Figure 3a) highlighted “tight junction,” “focal adhesion,” and “PI3K-Akt signaling pathway,” all of which play a significant role in fibrosis. GO analysis suggests that genes are enriched in “cell differentiation,” “extracellular matrix,” and “actin filament binding,” which are essential for maintaining cellular structure and function during fibrosis (Figures 3b, 3c, and 3d).

### 3.4. Correlation Analysis of Glycolytic Genes and Top 20 Associated Genes

**Figure 4:**
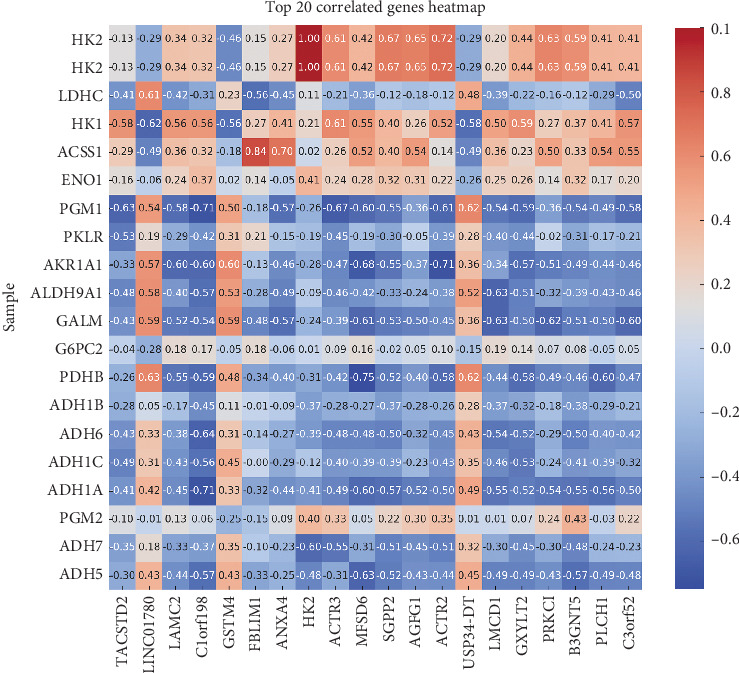
The correlation coefficients between the Top 10 glycolysis-related genes (*y*-axis) and various clinical biochemical traits (*x*-axis). HK2 demonstrates strong positive correlations with genes such as HK1 and LDHC. In contrast, negative correlations, particularly with PGM1 and G6PC2, suggest distinct roles in glucose metabolism.

During HSC activation, previous studies, as well as our own research, have indicated the presence of glucose metabolism disorders, particularly enhanced aerobic glycolysis. To investigate further, we analyzed whether genes associated with clinical traits regulate aerobic glycolysis and identified potential key regulators. Spearman correlation analysis was employed to investigate the connection between the RNA expression of 94 genes and those related to glycolysis and gluconeogenesis. HK2 showed significant positive correlations with both HK1 (*r* = 0.21) and LDHC (*r* = 0.11), indicating potential functional interactions or coregulation within the glycolytic pathway. In contrast, genes like PGM1 and G6PC2 showed negative correlations with glycolytic genes, indicating their potential opposing roles in cellular metabolism (Figure 4).

### 3.5. Correlation Between Top 10 Glycolysis-Related Genes and Clinical Traits

**Figure 5:**
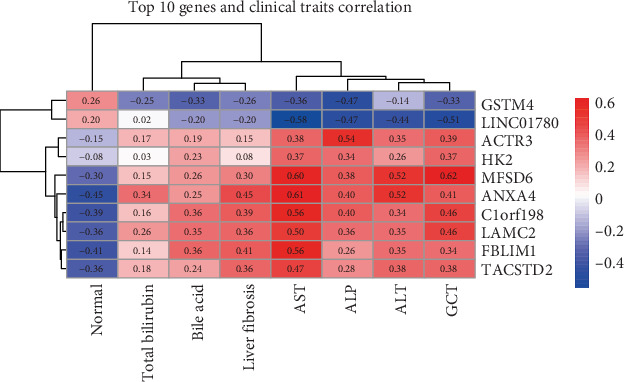
The correlation coefficients between the Top 10 glycolysis-related genes (*y*-axis) and various clinical biochemical traits (*x*-axis). The heatmap includes normal status, total bilirubin, bile acid, liver fibrosis, AST, ALP, ALT, and GGT levels.

Subsequently, the 10 genes most closely associated with glycolytic genes were chosen for Spearman correlation analysis with clinical traits. Importantly, a significant positive correlation was observed between HK2 and hepatic fibrosis (*r* = 0.08), as well as alanine aminotransferase (*r* = 0.26) and aspartate aminotransferase (*r* = 0.37). This indicates that HK2 may play a role in hepatic injury and fibrosis (Figure 5). Additionally, MFSD6 and ANXA4 were positively correlated with ALP, ALT, and hepatic fibrosis, indicating their roles in liver function. In contrast, the gene LINC01780 was negatively correlated with bile acids and GGT (Figure 5).

### 3.6. Construction of a Liver Fibrosis Predictive Model Using Five Machine Learning Algorithms

**Figure 6:**
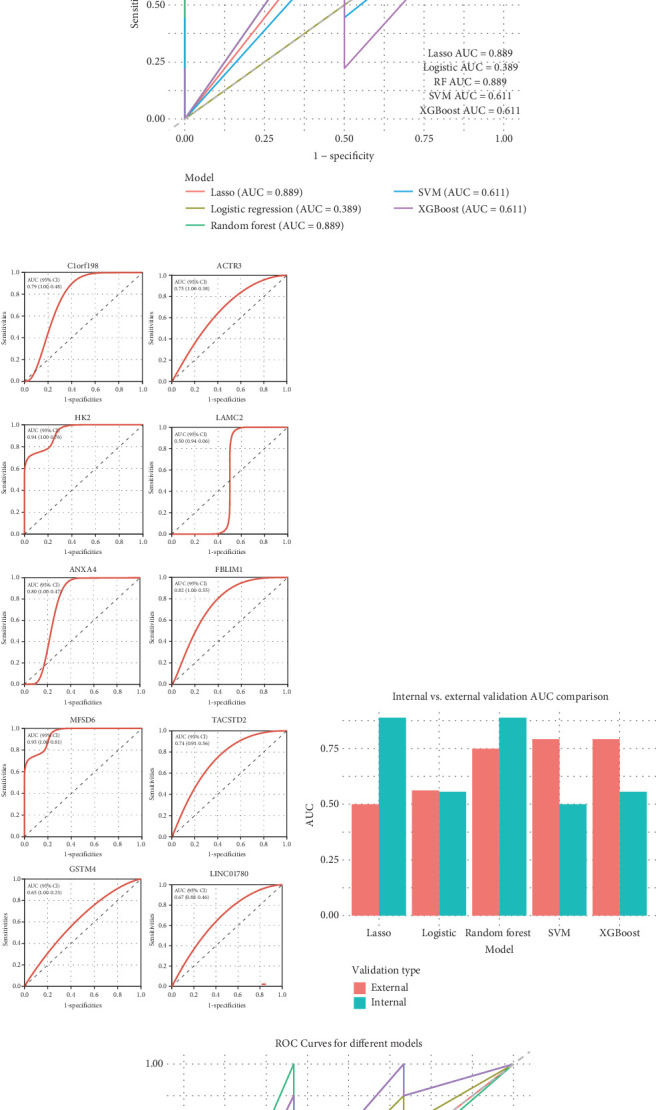
The performance of different machine learning models in predicting liver fibrosis. (a) The Lasso model cross-validation plot shows the selection of the optimal *λ* value through cross-validation, balancing model complexity and performance by minimizing binomial deviance. (b) Receiver operating characteristic (ROC) curves depict the performance of different machine learning models using internal dataset validation. Lasso regression and random forest models achieved the highest area under the curve (AUC = 0.889), while the logistic regression model exhibited the lowest AUC (0.389). SVM and XGBoost models demonstrated moderate performance with AUCs of 0.611. (c) ROC curves highlight the predictive power of individual genes for liver fibrosis, with genes such as LAMC2, HK2, and TACSTD2 showing strong predictive ability with high AUC values. (d) A comparison of AUC values between internal and external validation sets reveals that the random forest model maintained robust performance across both, while the logistic regression model experienced a notable drop in external validation. (e) ROC curves for external dataset validation indicate that the random forest model (AUC = 0.775) and XGBoost model (AUC = 0.792) exhibited the best predictive performance, while Lasso regression (AUC = 0.5) and logistic regression (AUC = 0.582) underperformed.

The Top 10 glycolysis-related genes were selected based on their strongest correlations with glycolysis genes for predicting liver fibrosis outcomes. Cross-validation of the Lasso model identified the optimal *λ* value, ensuring effective feature selection without overfitting (Figure 6a). The ROC curve analysis revealed that the Lasso and random forest models outperformed the logistic regression model, with AUC values of 0.889. The SVM and XGBoost models displayed moderate performance, each with an AUC of 0.611 (Figure 6b). Individual gene contributions were evaluated, with LAMC2, HK2, and TACSTD2 emerging as key contributors to model accuracy (Figure 6c).

External validation using GSE197112 and GSE252249 datasets confirmed the robustness of the random forest model (AUC = 0.775) and the strong performance of the XGBoost model (AUC = 0.792), while the Lasso and logistic regression models underperformed with AUCs of 0.5 and 0.582, respectively (Figure 6e). Comparison of five machine learning methods suggests that random forest trees perform best in terms of predictive ability in both internal and external validations (Figure 6d).

### 3.7. Pathways Affected by HK2 KO in HSCs

**Figure 7:**
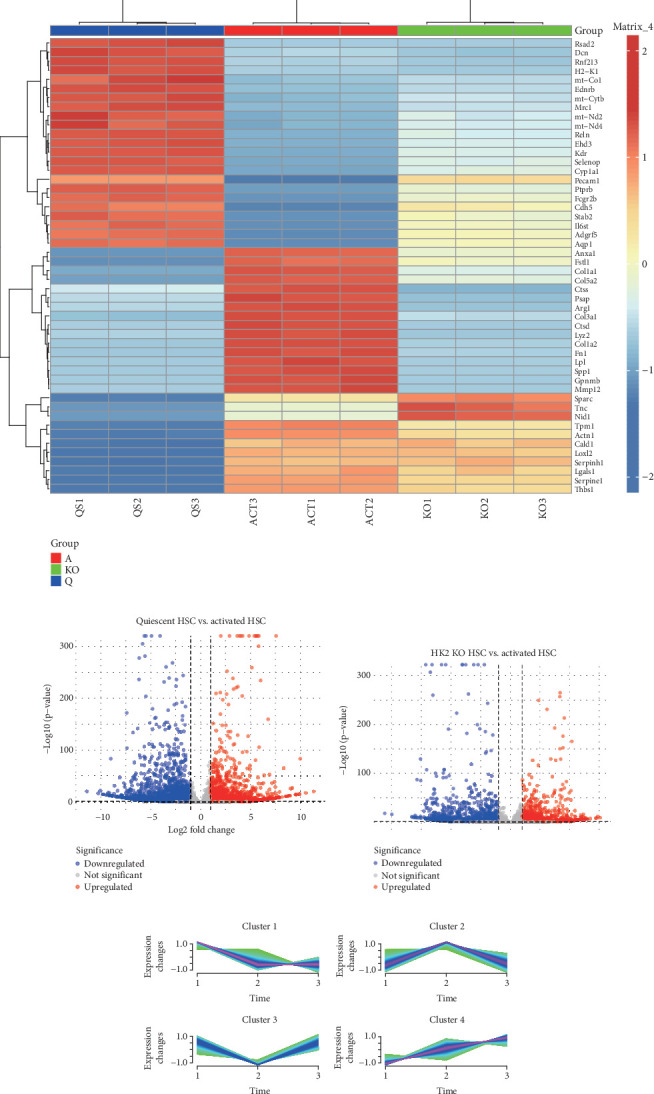
Examination of HSC RNA sequencing outcomes in diverse groups. (a) Principal component analysis (PCA) of gene expression data reveals clear separation among the three groups, with PC1 accounting for 80.80% of the variance, indicating distinct gene expression profiles. (b) A heatmap of the Top 50 differentially expressed genes across the groups shows red for upregulation and blue for downregulation. Significant clustering differences are observed between the activated and HK2 KO groups compared to the control. (c) The volcano plot shows the differences in gene expression between the quiescent HSC and activated HSC groups. Red dots represent genes with significant differences in expression (*p* < 0.05). (d) The volcano plot shows the differences in gene expression between the activated HSC and HK2 KO HSC groups. Red dots represent genes with significant differences in expression (*p* < 0.05). (e) Mfuzz clustering analysis identifies four distinct expression patterns (Clusters 1–4) over time, with each line representing gene expression dynamics for each cluster. (f) A Venn diagram shows the overlap of differentially expressed genes identified in three analyses: Mfuzz clustering, A versus Q comparison, and KO versus A comparison. The overlap highlights 1081 common genes between KO versus A and A versus Q, suggesting key regulatory pathways influenced by HK2 knockout. A = activation.

**Figure 8:**
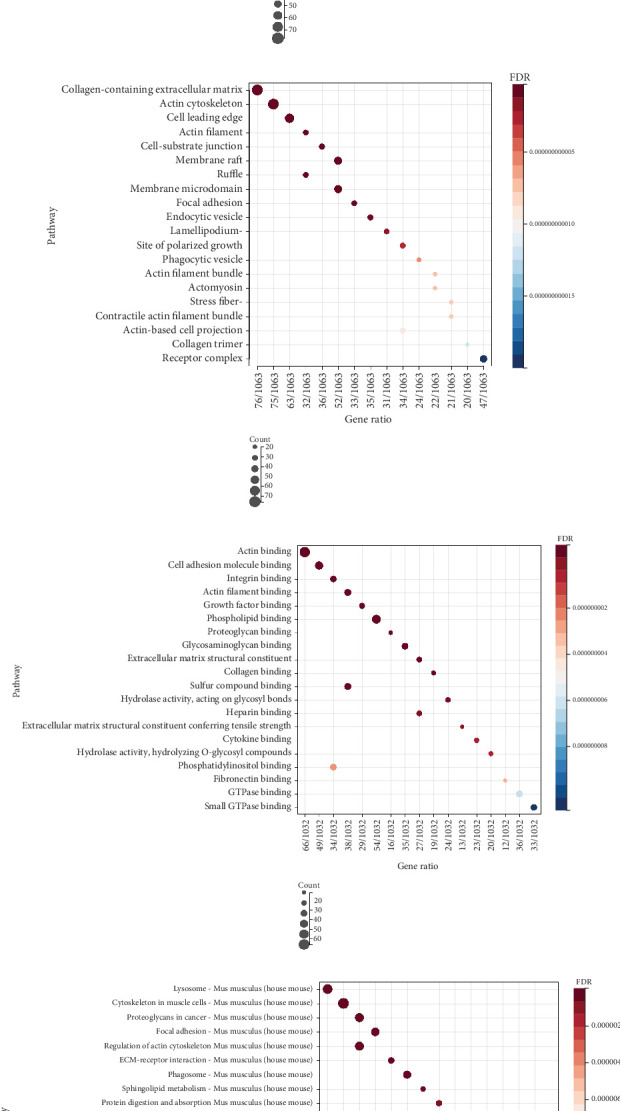
GO and KEGG enrichment analysis of differentially expressed genes following HK2 knockout. (a) Gene Ontology (GO) biological process (BP) enrichment analysis of genes identified from the intersection of Mfuzz clusters and differentially expressed genes reveals significant pathways. (b) GO cellular component (CC) enrichment analysis. (c) GO molecular function (MF) enrichment analysis shows enrichment in molecular functions. (d) KEGG pathway enrichment analysis identifies significant pathways.

For a more in-depth exploration of the pathways impacted by HK2, we acquired RNA-Seq data from HSC control, activation, and HK2 KO groups from GEO (GSE229154). Transcriptomic changes and affected pathways were analyzed. PCA showed notable distinctions among the control, activation, and HK2 KO groups, with PC1 explaining 80.80% of the variability (Figure 7). The heatmap of the top 50 DEGs (Figure 7) indicated that both the activation and KO groups had significant transcriptomic changes compared to the control group. The volcano plot (Figure 7) further highlighted significantly up- and downregulated genes, demonstrating the extensive impact of HK2 KO on the transcriptome. Mfuzz clustering identified four distinct expression clusters (Figure 7), with Cluster 2, which shared a similar expression pattern to HK2, selected for further analysis. A total of 1081 genes were commonly altered through the intersection with DEGs (Figure 7). Subsequently, the genes were analyzed for GO enrichment and KEGG pathway analysis (Figures 8, 8, and 8).

GO enrichment analysis of DEGs revealed that knocking out HK2 caused substantial changes in biological processes, cellular components, and molecular functions (Figures 8a, 8b, and 8c). The results showed that the differentially expressed proteins were mainly enriched in biological processes such as cell–matrix adhesion, actin, and ECM remodeling, suggesting that they may participate in pathological processes by regulating cell structure and signal transduction (Figures 8a, 8b, and 8c). The enrichment of DEGs in pathways such as focal adhesion and ECM-receptor interactions indicates that HK2 KO has a notable effect on ECM deposition, which is important in the development of liver fibrosis (Figure 8d).

### 3.8. HK2 Expression Differences Across Cell Types Revealed by Single-Cell Analysis

**Figure 9:**
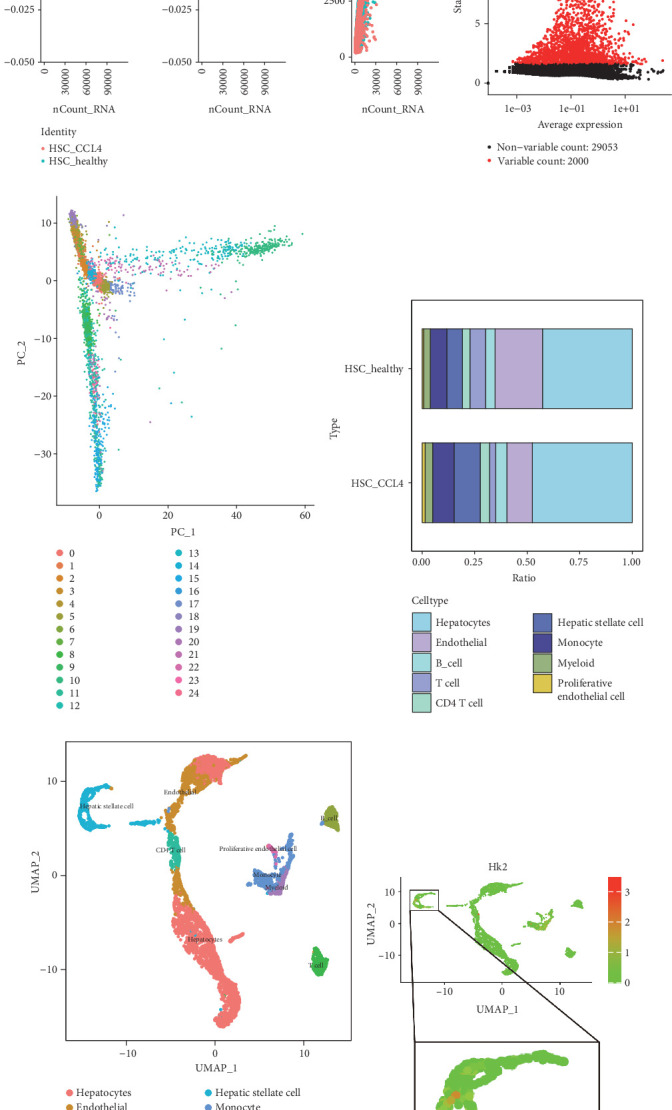
Single-cell RNA sequencing analysis. (a) The quality control data, specifically showing the correlation of housekeeping genes. The first plot indicates NA for correlation, which confirms that the sequencing data quality is sufficient. (b) Identifies highly variable genes across the dataset, highlighting genes such as Cxcl2 and Gm26767 as significantly variable. (c) Visualizes a principal component analysis (PCA) plot, demonstrating the clustering of various cell populations. (d) Depicts the differences in cell type composition between healthy hepatic stellate cells (HSCs) and fibrotic HSCs (HSC_CCL4), indicating an increased ratio of HSCs in fibrosis. (e) This UMAP plot highlights distinct cell populations, such as hepatocytes and HSCs. (f) Visualizes HK2 expression on the UMAP plot, confirming upregulation primarily in HSCs.

To further analyze the expression differences of HK2 in various liver cells, single-cell RNA sequencing datasets from the GEO database were downloaded and analyzed. The datasets encompassed one healthy mouse and one liver fibrosis mouse. Following data integration and rigorous quality control (Figure 9a), 2000 highly variable genes were identified (Figure 9b). Using the SingleR R package and manual annotation, PCA identified 24 unique clusters. The study included hepatocytes, liver sinusoidal endothelial cells, B cells, T cells, CD4 T cells, HSCs, neutrophils, myeloid-derived cells, and proliferating epithelial cells (Figure 9c). The analysis demonstrated an increased proportion of HSCs in the liver fibrosis mouse's single-cell data (Figure 9d). Further annotation on the UMAP plot, focusing on HK2 and related glycolysis genes, revealed that HK2 expression was predominantly localized within HSCs (Figure 9e,f).

### 3.9. Elevated HK2 Expression in Liver Fibrosis Tissues

**Figure 10:**
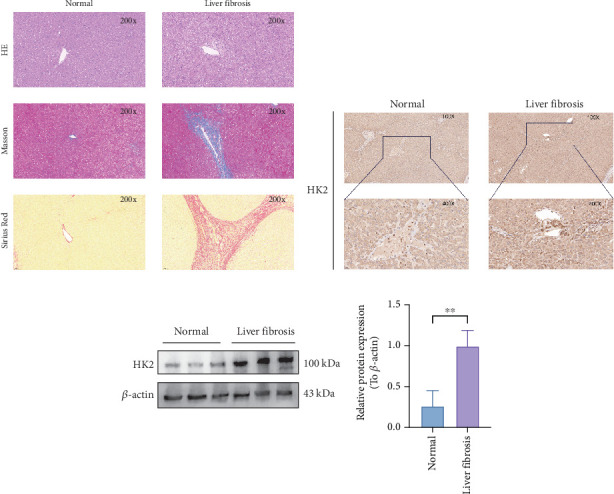
Histopathological and HK2 expression analysis in normal and fibrotic human liver tissues. (a) Representative images of human liver tissue sections stained with hematoxylin and eosin (HE), Masson's trichrome, and Sirius Red. The images show normal liver tissue and liver fibrosis tissue at 200× magnification. Masson's trichrome and Sirius Red staining highlight the increased collagen deposition in liver fibrosis tissues. (b) Immunohistochemical staining of HK2 in human liver tissues. Images at 100× and 400× magnification show increased HK2 expression in liver fibrosis tissues, particularly in regions consistent with hepatic stellate cells. (c) Western blot (WB) analysis of HK2 expression in human liver tissues, comparing normal and liver fibrosis samples. The adjacent bar graph shows densitometric analysis, indicating a significant increase in HK2 expression in liver fibrosis tissues (*p* < 0.01) relative to *β*-actin as the loading control. Data are expressed as mean ± SD. ⁣^∗∗^*p* < 0.01.

Histopathological examination of normal and fibrotic liver tissues was conducted using HE, Masson, and Sirius Red staining (Figure 10a). Fibrotic liver tissue showed pronounced fibrosis, marked by extensive collagen deposition, compared to normal liver tissue. A significant increase in HK2 expression was observed in fibrotic liver tissues, especially in the portal and periportal regions where HSC activation is predominant (Figure 10b).

To confirm differences in HK2 expression, WB was used to compare HK2 protein levels in normal and fibrotic liver tissues (see Figure 10c). The results showed that compared with normal tissue, HK2 expression was significantly upregulated in fibrotic liver tissue (*p* < 0.05), indicating that HK2 may be associated with the progression of liver fibrosis.

### 3.10. Elevated HK2 Expression in Liver Fibrosis Model Mice

**Figure 11:**
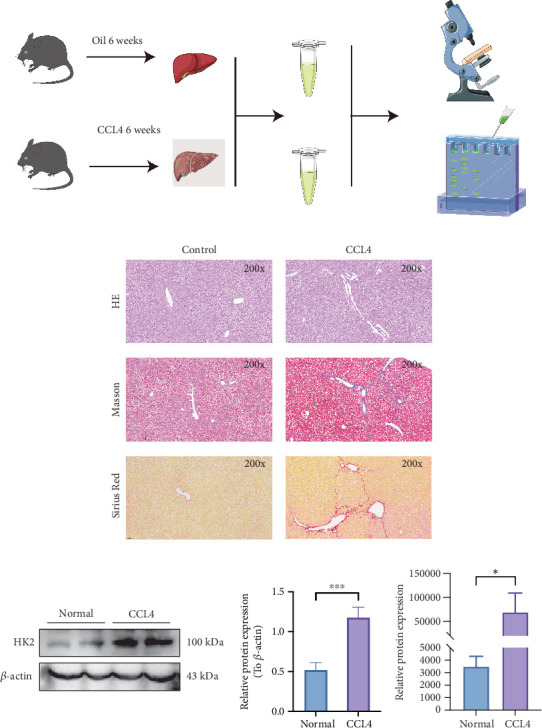
Histological and molecular analysis of HK2 in CCL4-induced liver fibrosis in mice. (a) Schematic representation of the experimental design for CCL4-induced liver fibrosis in mice. Control mice were treated with oil for 6 weeks, while the model group was treated with CCL4 for 6 weeks. Liver tissues were harvested for histological and molecular analysis. (b) Representative images of liver tissue sections from control and CCL4-treated mice stained with hematoxylin and eosin (HE), Masson's trichrome, and Sirius Red at 200× magnification. The images demonstrate increased collagen deposition and fibrosis in the CCL4-treated group. (c) Western blot analysis of HK2 expression in liver tissues from control and CCL4-treated mice. The adjacent bar graph shows densitometric analysis, indicating a significant increase in HK2 expression in the CCL4-treated group (*p* < 0.001). (d) Proteomic analysis of HK2 expression in liver tissues from control and CCL4-treated mice. The bar graph shows a significant increase in HK2 expression in the CCL4-treated group (*p* < 0.05). Data are expressed as mean ± SD. ⁣^∗^*p* < 0.05 and ⁣^∗∗∗^*p* < 0.001.

Liver fibrosis was induced in mice via CCl_4_ administration. Histopathological analysis of liver tissue was performed using HE staining, Masson staining, and Sirius Red staining (Figure 11a,b). Compared to the control group, the CCl_4_-treated mice exhibited pronounced fibrosis and marked collagen accumulation.

In the CCl_4_-treated group, WB analysis of liver tissues indicated a notable increase in HK2 expression when compared to controls (*p* < 0.001, Figure 11c). Additionally, proteomic analysis confirmed a significant elevation of HK2 in the liver fibrosis model (*p* < 0.05, Figure 11d). The involvement of HK2 in CCl_4_-induced liver fibrosis highlights its significance as a key player closely linked to the development of liver fibrosis.

### 3.11. HK2 Knockdown Impairs Wound Healing and Cell Migration

**Figure 12:**
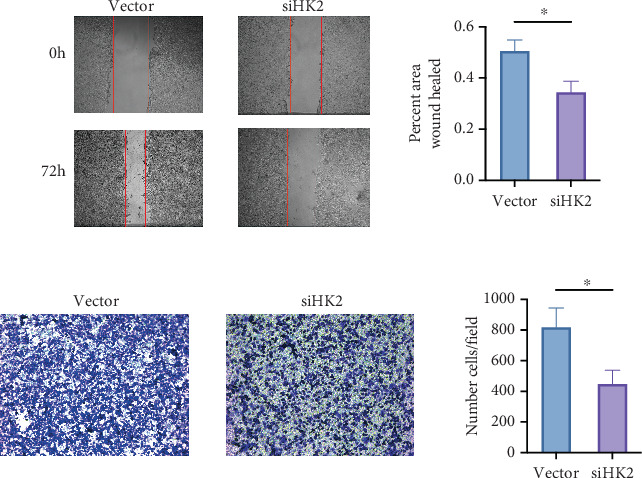
Impact of HK2 knockdown on cell migration and wound healing. (a) Wound healing assay comparing control (vector) and siHK2-treated cells at 0 and 72 h. Red lines mark the wound edges. Cells transfected with siHK2 exhibit significantly slower wound closure compared to the vector control at 72 h. The bar graph quantifies the percentage of wound area healed after 72 h, with siHK2-treated cells showing a significant reduction in healing capacity (*p* < 0.05). (b) Transwell migration assay results show that fewer cells migrated through the Transwell membrane in the siHK2 group compared to the vector group. The bar graph quantifies the number of migrated cells per field, demonstrating a significant decrease in migration in the siHK2 group (*p* < 0.05). Data are expressed as mean ± SD. ⁣^∗^*p* < 0.05.

The effects of HK2 knockdown on wound healing and cell migration were determined using in vitro assays by assessing functional outcomes. The ability of cells to heal wounds was significantly lower in those treated with siHK2 than in vector controls (Figure 12a). The siHK2 group exhibited a notably lower wound closure percentage than the control group after 72 h, suggesting impaired cell migration and proliferation (*p* < 0.05). The Transwell migration assay (Figure 12b) was employed to further assess cell migration, showing that the knockdown of HK2 led to a significant reduction in migrated cells compared to the controls, as quantified in the bar graph (*p* < 0.05). These results suggest that HK2 is crucial for cellular migration and wound healing, key processes in tissue repair and potentially linked to fibrosis progression.

## 4. Discussion

Liver fibrosis is a response to chronic liver injury, indicating an early and potentially reversible phase of cirrhosis [23]. Despite its reversibility, no effective pharmacological treatments are currently available [24]. Recent research has highlighted that activated HSCs adopt a metabolic phenotype characterized by aerobic glycolysis [13, 25]. This metabolic reprogramming provides energy for cell proliferation and function while exacerbating liver fibrosis through the excessive accumulation of extracellular matrix [26]. In this study, we used transcriptomics and single-cell transcriptomics to comprehensively investigate the expression of the HK2 gene in liver fibrosis, its relevance to the clinic, and the possible pathways affected by the HK2 gene at the human, animal, and cellular levels. Additionally, HK2 modulates the migratory capacity of LX-2 cells, as demonstrated by WB and immunohistochemistry analyses in both murine and human liver tissues. The results of our research provide insight into the molecular mechanisms that play a role in liver fibrosis and suggest potential targets for future therapeutic strategies.

In aerobic glycolysis, HK2 is crucial for initiating glycolysis and producing metabolic intermediates [27, 28]. As a key regulator of cellular energy metabolism, HK2 has gained considerable attention in the context of liver fibrosis [16]. Transcriptomic sequencing analysis demonstrated a significant elevation in HK2 expression in liver fibrosis tissues in our study. Previous research has shown that HK2 is significantly related to the development of several cancer types [29, 30]. It was observed that HK2 showed a significant increase in the liver fibrosis group when compared to the control group. A significant correlation with clinical biochemical indicators, including GGT, ALT, ALP, and AST, suggests a close link between HK2 expression and liver damage. This study preliminarily validated the effectiveness of HK2 in predicting disease progression, suggesting that HK2 might serve as a clinical biomarker. Additionally, recent translational research has underscored the importance of HK2 in liver cancer [31] and gastric cancer treatment [32], revealing its ability to induce resistance to targeted drugs. Although significant challenges remain, these findings suggest that targeted therapies against HK2 hold promise for clinical application.

RNA sequencing and enrichment analyses revealed significant enrichment of pathways related to tight junctions, focal adhesion, and ECM-receptor interactions, which are closely linked to cell migration, extracellular matrix remodeling, and adhesion. Furthermore, HK2 KO significantly affected lipid metabolism and lysosomal function pathways, highlighting its role in regulating cellular metabolism and autophagy. These findings highlight HK2's potential as a therapeutic target in liver fibrosis and its broader role in liver disease progression, aligning with previous reports [16].

Recent research has demonstrated that activated HSCs significantly upregulate aerobic glycolysis to facilitate their transformation into fibrogenic effector cells [13]. This study corroborates these findings by showing a significantly higher proportion of HSCs in mice with liver fibrosis compared to normal mice, as confirmed through single-cell analysis. Significant changes were observed in the expression levels of glycolytic enzymes, especially the upregulation of HK2 in HSCs. Previous studies have also indicated that HK2 exacerbates fibrosis by promoting the release of fibrotic nodules around the liver's central veins [16]. Targeted knockdown of HK2 in HSCs can significantly reduce liver fibrosis [16], aligning with our findings. However, previous studies have mainly focused on mice [16], and whether the same holds true in human tissues has not been investigated. Instead, we analyzed the sequencing data of human tissues through a combination of multiomics approaches. This offers the potential for clinical translation in the treatment of liver fibrosis.

Previous studies have indicated that HK2 boosts aerobic glycolysis in HSCs, indicating its involvement in the advancement of liver fibrosis [26]. In LX-2 cells, knocking down HK2 with siRNA inhibited migration, indicating that HK2 promotes HSC activation. Previous studies indicate that the HK2 gene boosts lactate production, causing genomic protein lactylation during HSC activation [16]. This process enhances the transcription of genes such as COL1A1, further activating HSCs. Additionally, according to our GO and KEGG analyses, the altered pathways are enriched in pathways associated with HSC activation. We hypothesize that HK2 produces significant lactate, influencing ECM deposition, cell adhesion, and other pathways through lactylation, thereby promoting liver fibrosis.

However, this study also has limitations. Although our results suggest that HK2 has a diagnostic efficacy of approximately 0.94, our sample size was small and only included samples from a single hospital. In addition, due to missing data, we were unable to compare our results with traditional liver fibrosis scoring systems. We also did not compare it with traditional liver fibrosis markers such as HA and LN. The role of other glycolytic enzymes in HSC activation has not yet been thoroughly investigated. Together with findings from a recent publication by our group, the observed alterations in glycolytic enzymes appear to influence HSC activation, highlighting the need for further exploration in future studies [33]. Although enrichment analysis identified multiple metabolic and signaling pathways associated with HK2, the interactions and regulatory networks among these pathways remain unclear. The cross-regulation between different pathways and the upstream–downstream relationships is not yet well defined. Future studies could employ systems biology or network analysis methods to further elucidate the interactions among these pathways and clarify the regulatory role of HK2 within these complex networks.

## 5. Conclusion

To summarize, this research emphasizes the crucial importance of HK2 in the metabolic changes linked to liver fibrosis, especially in the stimulation of HSCs. With its participation in glycolysis and its ability to affect cellular energy dynamics, HK2 appears to be a promising target for treating liver fibrosis.

## Data Availability

The datasets generated and analyzed during this study are available from the corresponding author, Xueke Zhao, upon reasonable request. The RNA sequencing data and related transcriptomic analyses can be accessed from the GEO database (https://www.ncbi.nlm.nih.gov/geo/) under Accession Numbers GSE197112, GSE252249, GSE139602, and GSE175939. Additional data supporting the findings of this study are also available from the authors upon reasonable request. For data requests, please contact the corresponding author, Xueke Zhao.

## Supporting Information

Supporting Information Additional supporting information can be found online in the Supporting Information section. Table S1: Changes in biochemical indices in normal and liver fibrosis groups. Values are median (Q1 and Q3) for continuous variables and *n* (%) for categorical variables. Data were analyzed using the Mann–Whitney *U* test (rank-sum test) for continuous variables.
