# Supplementary material for: Integrative Multiomics Analysis Identifies HK2 as a Key Regulator of Metabolic Reprogramming in Hepatic Stellate Cells
Source: Hum Mutat. 2025 Nov 7;2025:1584910. doi: 10.1155/humu/1584910 (PMC12618131; doi:10.1155/humu/1584910)
Supplement: Supporting Information — Additional supporting information can be found online in the Supporting Information section. Table S1: Changes in biochemical indices in normal and liver fibrosis groups. Values are median (Q1 and Q3) for continuous variables and n (%) for categorical variables. Data were analyzed using the Mann–Whitney U test (rank-sum test) for continuous variables. [file 1584910.f1.docx]

**Table S 1 Changes in biochemical indices in normal and liver fibrosis groups**

| variable | Totle（n=38） | groups | | *P value* |
| --- | --- | --- | --- | --- |
| sex, n (%) |  | Liver fibrosis  (n = 30) | control  (n = 8) | 0.689 |
| Females | 13 (34.21) | 11 (36.67) | 2 (25.00) |  |
| Males | 25 (65.79) | 19 (63.33) | 6 (75.00) |  |
| Age, M (Q₁, Q₃) | 53.76±7.4 | 54.6±9.49 | 52.12±14.01 | 0.897 |
| bile acids, M (Q₁, Q₃) | 9.46 (3.26 - 36.03) | 16.00 (3.63 - 59.65) | 4.67 (2.38 - 6.54) | 0.019 |
| ALT, M (Q₁, Q₃) | 25.65 (17.83 - 43.88) | 28.30 (20.42 - 49.48) | 14.85 (12.62 - 22.85) | 0.013 |
| AST, M (Q₁, Q₃) | 29.30 (23.83 - 47.20) | 35.80 (26.70 - 51.18) | 19.80 (17.08 - 23.90) | 0.001 |
| Total Bilirubin, M (Q₁, Q₃) | 11.61 (7.10 - 20.02) | 16.02 (9.12 - 20.80) | 7.30 (5.56 - 10.10) | 0.025 |
| Direct Bilirubin, M (Q₁, Q₃) | 4.40 (2.80 - 7.61) | 5.55 (3.50 - 8.82) | 2.65 (1.94 - 3.38) | 0.006 |
| Indirect Bilirubin, M (Q₁, Q₃) | 6.75 (4.20 - 11.37) | 7.88 (5.03 - 13.78) | 4.61 (3.91 - 7.03) | 0.082 |
| GGT, M (Q₁, Q₃) | 51.96 (29.18 - 83.61) | 55.98 (35.66 - 128.70) | 34.43 (14.99 - 58.93) | 0.070 |
| ALP, M (Q₁, Q₃) | 103.50 (91.00 - 136.25) | 108.00 (83.50 - 148.25) | 97.50 (92.75 - 104.50) | 0.316 |
| Viral Hepatitis B, M (Q₁, Q₃) | 1.00 (0.00 - 1.00) | 1.00 (0.25 - 1.00) | 0.00 (0.00 - 0.00) | 0.002 |

Values are median (Q1, Q3) for continuous variables and n (%) for categorical variables. Data were analyzed using the Mann-Whitney U test (rank-sum test) for continuous variables.
